# Gestational Age‐Dependent Effects of Antenatal Magnesium Sulfate on Fetal S100B Levels: An Observational Study Using Cord Serum

**DOI:** 10.1111/jog.70208

**Published:** 2026-02-15

**Authors:** Takuma Shimaya, Kazuya Fuma, Sho Tano, Seiko Matsuo, Takafumi Ushida, Kenji Imai, Hiroaki Kajiyama, Tomomi Kotani

**Affiliations:** ^1^ Department of Obstetrics and Gynecology Nagoya University Graduate School of Medicine Nagoya Japan; ^2^ Division of Reproduction and Perinatology, Center for Maternal‐Neonatal Care Nagoya University Hospital Nagoya Japan; ^3^ Department of Obstetrics and Gynecology Hamamatsu University School of Medicine Hamamatsu Japan

**Keywords:** fetal neuroprotection, magnesium sulfate, preterm birth, S100B, umbilical cord blood

## Abstract

**Aim:**

Magnesium sulfate (MgSO_4_) is widely used for fetal neuroprotection in preterm births before 32 weeks of gestation, yet it remains unclear whether its effect depends on gestational age. S100 calcium‐binding protein B (S100B), a protein secreted by astrocytes, is recognized as a biomarker of neural distress. This study aimed to investigate the relationship between antenatal MgSO_4_ administration and umbilical cord serum S100B levels, with a focus on gestational age.

**Methods:**

This retrospective study included women who delivered between 22^+0^ and 33^+6^ weeks of gestation at a tertiary center. Patients with hypertensive disorders of pregnancy, category 1 cesarean sections, multiple pregnancies, major congenital anomalies, or insufficient MgSO_4_ administration were excluded. Cord blood samples were analyzed for S100B levels using ELISA. Multiple linear regression and restricted cubic spline modeling were performed to assess the association between MgSO_4_ and S100B levels across different gestational ages.

**Results:**

Among 69 eligible patients, MgSO_4_ administration was significantly associated with higher cord serum S100B levels who delivered at ≥ 30 weeks of gestation (adjusted estimate 0.39, 95% confidence interval 0.17–0.62), but not in those who delivered at < 30 weeks (−0.12, −0.43 to 0.19) in multiple linear regression adjusted for birth weight and antenatal corticosteroids. This association remained consistent across multiple sensitivity analyses. S100B levels exhibited a gestational age‐dependent increase in response to MgSO_4_, peaking at approximately 32 weeks in restricted cubic spline modeling.

**Conclusions:**

Antenatal MgSO_4_ administration beyond 30 weeks of gestation at delivery is associated with increased fetal S100B levels, suggesting a potential gestational age‐specific response.

## 1 Background

Preterm birth is a leading cause of neonatal death and complications. To date, only corticosteroids and magnesium sulfate administration have strong evidence as antenatal therapy for preventing brain damage in preterm [1]. A recent meta‐analysis demonstrated that magnesium sulfate compared with placebo reduced cerebral palsy up to 2 years' corrected age (risk ratio [RR] 0.71) and probably reduced severe intraventricular hemorrhage (Grade 3 or 4) (RR 0.76) [2]. Currently, many guidelines worldwide recommend magnesium sulfate administration for neuroprotection up to 32 or 34 weeks of gestation [3]. However, there is limited data about the optimal gestational age (GA) for magnesium sulfate use. Thus, the Magnesium Sulphate at 30 to 34 Weeks' Gestational Age (MAGENTA) trial [4] was conducted to investigate magnesium sulfate administration between 30 and 34 weeks of gestation; however, it failed to show significant improvement of neurodevelopmental outcomes at 2 years of age, including death or CP (adjusted RR 1.19, 95% confidence interval [CI] 0.65–2.18). In response to this result, the authors suggested that the mechanisms of brain injury in extremely preterm births may differ from those at later GAs. Furthermore, in secondary analysis of this study, children exposed to magnesium sulfate were more likely to be in the clinical problem range of anxiety (RR 2.14, 95% CI 1.18–3.88), withdrawal (RR 1.47, 95% CI 1.06–2.04), sleeping problem (RR 2.54, 95% CI 1.14–5.67), and total scores (RR 1.62, 1.00–2.64) in the Child Behavior Checklist [4]. Therefore, magnesium sulfate may have potentially negative effects on the fetal brain during this period, suggesting the need to reconsider its use in certain cases.

S100 calcium‐binding protein B (S100B) is a member of the S100 family of proteins and is known to be secreted mainly from astrocytes in the brain. S100B levels in biological fluids are recognized as one of the most reliable biomarkers of active neural distress, and a high concentration of S100B directly relates to the progress of several neural diseases [5, 6, 7]. A sudden increase in blood S100B levels is believed to reflect either a substantial release of S100B within the brain due to some form of stress, a disruption of the blood–brain barrier (BBB), or an increase in the permeability of the BBB to S100B [8]. Cord blood S100B levels have been reported to be associated with cerebral palsy [9] and could predict premature brain injury [10]. Postoperative S100B levels have been reported to be associated with neurodevelopmental outcomes at 2 years in children with congenital heart disease [11]. A recent study using mendelian randomization analysis concluded that increased postnatal S100B levels causally increase the risk of major depression disorder [12]. Therefore, cord serum S100B levels may serve as an objective parameter to evaluate the neuroprotective effects of antenatal magnesium sulfate. However, clinical evidence on the association between antenatal magnesium sulfate and fetal S100B levels is limited, with only one subgroup analysis reported in a case–control study [9].

To address this gap, this study aimed to investigate whether antenatal magnesium sulfate influences fetal S100B levels and whether this effect varies according to GA. Based on the findings of the MAGENTA trial [4], we stratified our analysis into two groups: those born before 30 weeks and those born at 30 weeks or later.

## 2 Method

### 2.1 Study Population

**FIGURE 1:**
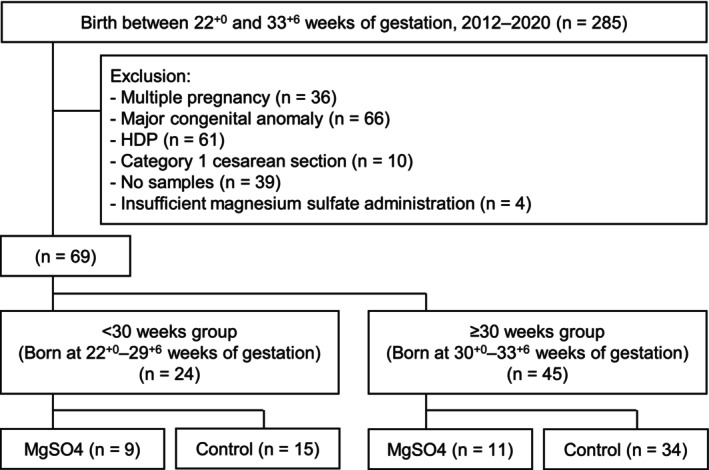
Participant flow diagram of the study population. This flowchart shows the selection process for the study population. Among 285 neonates born at 22^+0^–33^+6^ weeks of gestation (2012–2020), 69 were included after applying exclusion criteria. They were categorized into < 30 weeks at delivery (*n* = 24) and ≥ 30 weeks at delivery (*n* = 45) groups, and further divided into two groups: those who received antenatal magnesium sulfate and controls.

Data were retrospectively collected from the electric medical records system at Nagoya University Hospital, a tertiary referral center. Women who delivered between 22^+0^ and 33^+6^ weeks of gestation from January 2012 to December 2020 were included (Figure 1). Cases with hypertensive disorders of pregnancy (HDP) and Category 1 cesarean sections (defined as an immediate threat to the life of a woman or fetus requiring cesarean section within 30 min [13]) were excluded to minimize potential confounding when investigating the relationship between magnesium sulfate administration and its neuroprotective effects. In HDP cases, magnesium sulfate is commonly administered, while in Category 1 cesarean sections, it is typically absent. Both conditions could independently affect neurodevelopmental outcomes, complicating the interpretation of magnesium sulfate's specific effects. The other exclusion criteria were as follows: multiple pregnancy, major congenital anomaly, cases without cord serum samples, and insufficient magnesium sulfate administration (defined as a total dose < 4 g or discontinuation within 4 h before delivery, considering the drug's half‐life). HDP was diagnosed according to the definition by the International Society for the Study of Hypertension in Pregnancy in 2018 [14].

Clinical information was collected, including antenatal magnesium sulfate administration, maternal age at delivery, parity, antenatal corticosteroids, delivery mode, labor onset, GA at delivery, neonatal sex, birth weight, small for gestational age (SGA), Apgar score < 7 at 5 min after birth, umbilical artery pH < 7.1, histological chorioamnionitis (h‐CAM), and funisitis. The criteria of diagnosis were as follows. Labor onset was defined as regular, painful contractions accompanied by cervical changes [15]. h‐CAM was defined as Stage ≥ II according to the Blanc classification [16]; this threshold was selected because Stage II and Stage III chorioamnionitis have been reported to be associated with worsened neonatal outcomes [17]. SGA was defined as a birth weight below the 10th percentile on a Japanese growth chart [18].

The administration of magnesium sulfate started with or without a 4 g loading dose per 20 min and subsequently maintained at 1 g per hour intravenously via an infusion pump. Physicians determined the protocols of magnesium sulfate administrations according to the degree of uterine contraction or maternal side effects; therefore, the treatment periods and total amount of magnesium sulfate were various among patients. Patients who received magnesium sulfate before delivery were categorized in the MgSO_4_ group, and those who did not were categorized in the control group. Furthermore, the study population was categorized into the < 30 weeks group and the ≥ 30 weeks group (*n* = 45), according to the previous study [4].

### 2.2 Measurements of Cord Blood

Umbilical venous cord blood samples were collected immediately after delivery and stored at 4°C, after which the serum of the blood samples was separated by centrifugation and stored at −80°C until analysis. Cord serum magnesium concentrations were measured using a chelation quantification kit MG01M (Metallogenics Co. Ltd., Chiba, Japan). S100B and IL‐6 levels were measured using commercial Enzyme‐Linked Immunosorbent Assay (ELISA) kit YK151 (Yanaihara Institute Inc., Shizuoka, Japan) and D6050 (R&D Systems, Minneapolis, USA), respectively. ELISA samples were measured in duplicates, and the mean values were used for the analyses. “High magnesium level” was defined as a magnesium concentration above the 90th percentile of the control group. Due to insufficient blood samples, two cases from the magnesium sulfate group and one case from the control group were excluded from the IL‐6 analysis.

### 2.3 Statistical Analysis

We conducted the statistical analysis using R (version 4.2.3). Fisher's exact test or the Mann–Whitney *U* test was used for univariate analysis of categorical variables or numerical variables, respectively. The distribution of numerical data was presented as median (IQR). In a multiple linear regression analysis for cord serum S100B levels (ng/mL) between groups, multiple models were used as sensitivity analyses. The covariates for each model were as follows: Model 1 included birth weight and antenatal corticosteroids; Model 2 included birth weight, antenatal corticosteroids, and labor onset; Model 3 included birth weight, antenatal corticosteroids, and h‐CAM; Model 4 included birth weight, antenatal corticosteroids, and IL‐6 (log‐transformed). Log‐transformed IL‐6 levels were used because of a positively skewed distribution [19]. We included birth weight and antenatal corticosteroids as covariates in all models because they have been reported to be associated with S100B levels [20, 21]. To evaluate the differences in the effect of magnesium sulfate on S100B levels between GA groups, we performed an interaction analysis by comparing neonates born before 30 weeks of gestation (< 30 weeks group) with those born at 30 weeks or later (≥ 30 weeks group). A model including an interaction term was constructed, and the *p* value for the interaction was calculated. Statistical significance was defined as a *p* < 0.05.

### 2.4 Restricted Cubic Spline (RCS) Model Visualization

To model the nonlinear relationship between GA and S100B levels, we used RCS regression with four knots, implemented via the rms package in R (version 6.7‐1, https://CRAN.R‐project.org/package=rms), as applied in previous studies [22, 23]. An ordinary least squares (OLS) regression model was constructed with S100B levels as the dependent variable and magnesium sulfate administration, GA, antenatal corticosteroid administration (ACS), and birth weight as independent variables, as follows: S100B ~ magnesium sulfate administration × rcs(GA, 4) + ACS + birth weight (kg).

Adjusted estimates for magnesium sulfate administration (magnesium sulfate administration = 1) were obtained using the Predict function, with ACS set to 0 and Birth Weight fixed at the mean value. The estimates were visualized using ggplot2, with a 95% CI. The same approach was used for the sensitivity analysis with magnesium levels instead of magnesium sulfate administration.

## 3 Results

### 3.1 Eligible Patients and Characteristics

A total of 69 patients were eligible and categorized into the < 30 weeks group (*n* = 24) (MgSO_4_ group [*n* = 9], control group [*n* = 15]), and the ≥ 30 weeks group (*n* = 45) (MgSO_4_ group [*n* = 11], control group [*n* = 34]) based on gestational age at delivery and magnesium sulfate administration (Figure 1). There was no significant difference between the groups with the following variables: maternal age at delivery, parity, delivery mode, gestational age at delivery, antenatal corticosteroids administration, neonatal sex, birth weight, Apgar score < 7 at 5 min after birth, umbilical artery pH < 7.1, and SGA (Table 1). Cord serum levels of IL‐6 and the prevalence of funisitis were significantly higher in the MgSO_4_ group. The rates of labor onset and h‐CAM were not significantly different, but higher trends were observed in the MgSO_4_ group. Cord magnesium concentrations were significantly elevated in the MgSO_4_ group (*p* < 0.001). The both of 66.7% (< 30 weeks) and 81.8% (≥ 30 weeks) of neonates in the MgSO_4_ group had higher cord magnesium levels than the 90th percentile in the control group (2.56 mg/dL).

**TABLE 1:** Background characteristics of the study population.

Characteristic	< 30 weeks group		*p*	≥ 30 weeks group		*p*
	MgSO_4_	Control		MgSO_4_	Control	
	*n* = 9	*n* = 15		*n* = 11	*n* = 34	
Age (years)	32.0 [29.0, 35.0]	34.0 [31.0, 37.0]	0.11	34.0 [30.0, 39.0]	32.0 [27.2, 36.8]	0.27
Primigravida	4 (44.4)	6 (40.0)	1.00	6 (54.5)	16 (47.1)	0.93
ACS	7 (77.8)	13 (86.7)	1.00	9 (81.8)	24 (70.6)	0.73
CS	7 (77.8)	14 (93.3)	0.63	7 (63.6)	25 (73.5)	0.81
Labor onset	5 (55.6)	2 (13.3)	0.08	7 (63.6)	12 (35.3)	0.19
Gestational age at delivery (weeks)	27.4 [27.1, 27.9]	28.4 [27.6, 29.4]	0.11	32.4 [31.1, 33.5]	32.1 [31.5, 33.5]	0.95
Male	5 (55.6)	6 (40.0)	0.75	5 (45.5)	23 (67.6)	0.34
Birth weight (g)	992 [938, 1116]	1143 [894, 1344]	0.33	1664 [1295, 1982]	1769.5 [1596.5, 2019]	0.44
SGA	0 (0.0)	3 (20.0)	0.43	2 (18.2)	4 (11.8)	0.97
Apgar score < 7 at 5 min	5 (55.6)	6 (40.0)	0.75	3 (27.3)	6 (17.6)	0.80
Umbilical artery pH < 7.1^a^	0 (0.0)	0 (0.0)	NA	0 (0.0)	0 (0.0)	NA
h‐CAM	3 (33.3)	3 (20.0)	0.81	5 (45.5)	5 (14.7)	0.09
Funisitis	2 (22.2)	3 (20.0)	0.64	4 (36.4)	3 (8.8)	0.05
Cord IL‐6 levels (pg/mL)^b^	4.9 [3.3, 9.5]	1.0 [0.0, 4.8]	0.10	18.0 [5.3, 108.0]	0.7 [0.0, 8.6]	0.01
Cord magnesium levels (mg/dL)	3.2 [2.5, 3.4]	2.1 [1.9, 2.3]	0.01	3.8 [3.1, 4.2]	2.0 [1.9, 2.2]	< 0.001
High magnesium levels^c^	6 (66.7)	2 (13.3)	0.03	9 (81.8)	3 (8.8)	< 0.001
Total administration time of MgSO_4_ (h)	84.0 [48.0, 168.0]	0.0 [0.0, 0.0]	< 0.001	120.0 [25.5, 372.0]	0.0 [0.0, 0.0]	< 0.001
Total administration amount of MgSO_4_ (g)	84.0 [38.4, 172.8]	0.0 [0.0, 0.0]	< 0.001	120.0 [25.5, 368.4]	0.0 [0.0, 0.0]	< 0.001

*Note:* Fisher's exact test, *n* (%); Mann–Whitney *U* test, median [IQR].

Abbreviations: ACS, antenatal corticosteroids; CS, cesarean section; h‐CAM, histological chorioamnionitis; SGA, small for gestational age.

^a^ Umbilical cord pH value was missing in one case in the control group (≥ 30 weeks at delivery).

^b^ IL‐6 values were missing in three cases due to limited sample volume: the MgSO_4_ group (≥ 30 weeks at delivery), *n* = 1; the control group (< 30 weeks at delivery), *n* = 1; the control group (≥ 30 weeks at delivery), *n* = 1.

^c^ Higher than a 90th percentile in the control group (2.56 mg/dL).

### 3.2 S100B Levels in Umbilical Cord Blood

**FIGURE 2:**
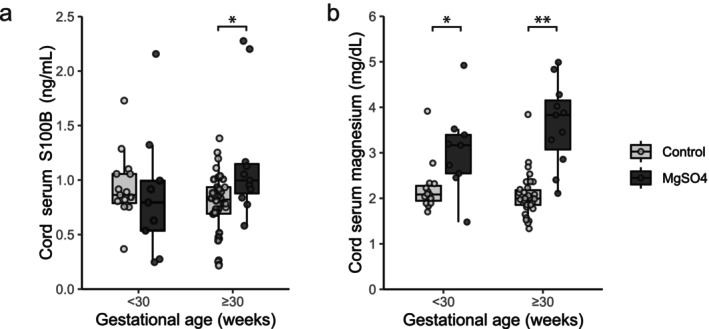
Cord blood S100B and magnesium levels in MgSO_4_ and control groups across gestational age at delivery. (a) Cord serum S100B and (b) magnesium levels in neonates. Each dot corresponds to an individual case. Box, line, and whisker summarize interquartile range (IQR), median, and upper or lower limit in 1.5 × IQR, respectively. *p < 0.05, **p < 0.001, Mann–Whitney *U* test.

S100B levels were significantly higher in the MgSO_4_ group compared to the control group at ≥ 30 weeks (*p* = 0.02) but not at < 30 weeks (*p* = 0.47, Figure 2a). In contrast, cord blood magnesium concentrations were significantly higher in the MgSO_4_ group compared to the control group both at < 30 (*p* = 0.01) and ≥ 30 weeks (*p* < 0.001, Figure 2b).

The cord S100B levels were also compared between the MgSO_4_ and control groups for each categorized group (the < 30 and ≥ 30 weeks groups) with multiple linear regression analyses (Table 2). To assess the robustness of the findings, four different models were constructed, each adjusting for potential confounding factors: In the < 30 weeks group, magnesium sulfate administration was not significantly associated with cord S100B levels. However, in the ≥ 30 weeks group, the cord S100B levels were significantly higher in the MgSO_4_ group across all four models in the sensitivity analysis of covariates. Furthermore, the interaction term between magnesium sulfate administration and the gestational age group was statistically significant in all models.

**TABLE 2:** Effects of magnesium sulfate on S100B levels stratified by gestational age at delivery (< 30 vs. ≥ 30 weeks).

	Cord serum S100B levels (ng/mL)									
	Clude, B (95% CI)		Model 1, B (95% CI)		Model 2, B (95% CI)		Model 3, B (95% CI)		Model 4, B (95% CI)	
	< 30 weeks	≥ 30 weeks	< 30 weeks	≥ 30 weeks	< 30 weeks	≥ 30 weeks	< 30 weeks	≥ 30 weeks	< 30 weeks	≥ 30 weeks
	*n* = 24	*n* = 45	*n* = 24	*n* = 45	*n* = 24	*n* = 45	*n* = 24	*n* = 45	*n* = 23^a^	*n* = 43^a^
*Variable*										
MgSO_4_	−0.06 (−0.44, 0.32)	0.39 (0.13, 0.65)**	−0.12 (−0.43, 0.19)	0.39 (0.17, 0.62)**	−0.16 (−0.53, 0.21)	0.39 (0.15, 0.63)**	−0.11 (−0.43, 0.21)	0.31 (0.08, 0.55)**	−0.22 (−0.54, 0.11)	0.31 (0.04, 0.57)*
*Covariates*										
Birth weight (kg)			0.00 (−0.55, 0.55)	−0.29 (−0.55, −0.02)*	0.02 (−0.55, 0.59)	−0.29 (−0.56, −0.02)*	−0.06 (−0.65, 0.53)	−0.34 (−0.60, −0.08)*	−0.04 (−0.62, 0.54)	−0.34 (−0.62, −0.06)*
ACS			−0.71 (−1.12, −0.29)**	−0.35 (−0.57, −0.13)**	−0.74 (−1.21, −0.28)**	−0.35 (−0.57, −0.12)**	−0.69 (−1.12, −0.27)**	−0.38 (−0.60, −0.17)***	−0.85 (−1.32, −0.39)**	−0.39 (−0.62, −0.16)**
Labor onset					0.09 (−0.32, 0.49)	0.00 (−0.20, 0.21)				
h‐CAM							−0.12 (−0.49, 0.25)	0.26 (0.01, 0.50)*		
IL‐6 (log‐transformed)									0.02 (−0.05, 0.08)	0.02 (−0.01, 0.06)
*R* ^*2*^	0.00	0.18	0.41	0.41	0.41	0.41	0.42	0.47	0.50	0.44
*F*	*F*(1, 22) = 0.11	*F*(1, 43) = 9.40**	*F*(3, 20) = 4.54*	*F*(3, 41) = 9.60***	*F*(4, 19) = 3.32*	*F*(4, 40) = 7.03***	*F*(4, 19) = 3.43*	*F*(4, 40) = 8.95***	*F*(4, 18) = 4.43*	*F*(4, 38) = 7.33***
*p* for the interaction between gestational groups and MgSO_4_ estimate		0.04*		0.006**		0.006**		0.007**		0.007**

*Note:* The results of multiple linear regression analyses examining the association between antenatal magnesium sulfate administration and umbilical cord serum S100B levels, stratified by gestational age (< 30 vs. ≥ 30 weeks). Crude: The crude effects of antenatal magnesium sulfate administration on cord serum S100B levels. Model 1: Adjusted for birth weight and ACS. Model 2: Adjusted for birth weight, ACS, and labor onset. Model 3: Adjusted for birth weight, ACS, and h‐CAM. Model 4: Adjusted for birth weight, ACS, and log‐transformed IL‐6 level.

Abbreviations: ACS, antenatal corticosteroids; CI, confidence interval; h‐CAM, histological chorioamnionitis.

^a^ Number reduction due to limited sample.

* *p* < 0.05.

** *p* < 0.01.

*** *p* < 0.001.

We also investigated an association between cord S100B levels and magnesium levels instead of magnesium sulfate administration (Table S1). In the < 30 weeks group, high magnesium levels (> 90th percentile of the control group) were not significantly associated with S100B levels but showed a significant association in the ≥ 30 weeks group across all models in the sensitivity analysis of covariates. This association was consistent with the relationship observed between magnesium sulfate administration and S100B levels; however, the interaction with gestational groups was not significant (Table S1).

In addition, associations between some covariates and S100B levels were also observed: a covariate of ACS was significantly associated with lower S100B levels in all models; a covariate of birth weight was significantly associated with lower S100B levels in any models in the ≥ 30 weeks group; and a covariate of h‐CAM was significantly associated with higher S100B levels in the Model 3 in the ≥ 30 weeks group (Table 2). These associations were also shown in the results of the sensitivity analysis (Table S1).

### 3.3 Restricted Cubic Spline Visualization

**FIGURE 3:**
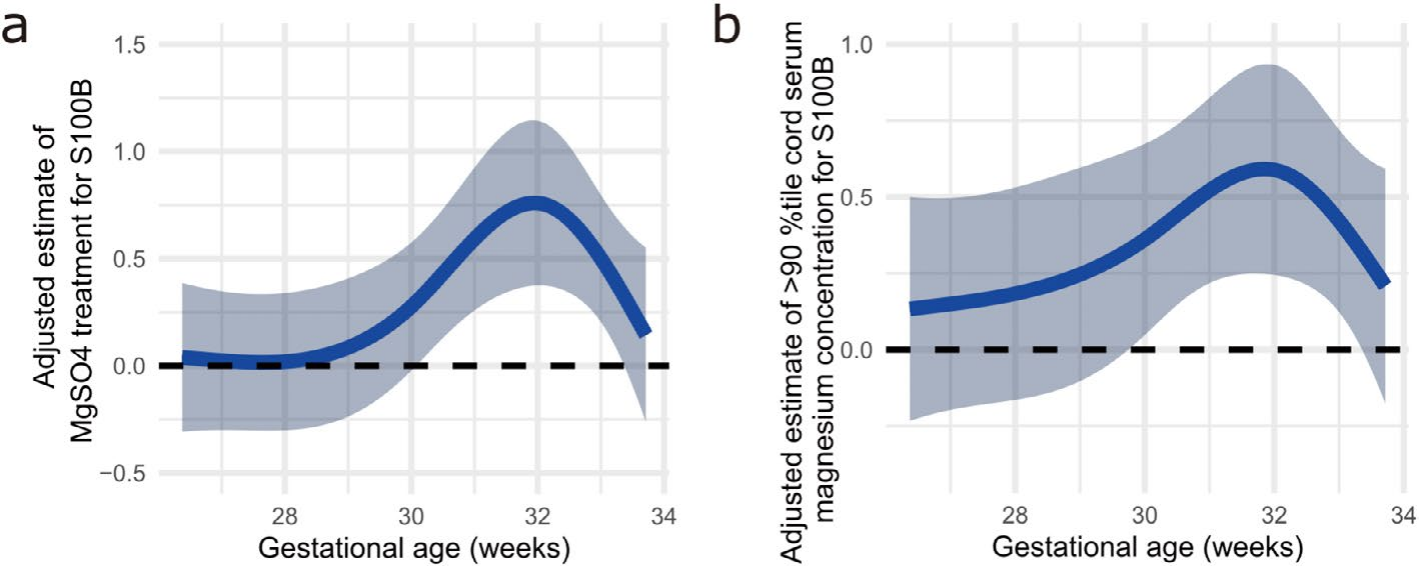
Restricted cubic spline visualization of the effect of magnesium sulfate and high magnesium levels on S100B across gestational age at delivery. Adjusted estimates for the effect of (a) magnesium sulfate administration and (b) high cord magnesium levels (> 90th percentile) on cord serum S100B levels were modeled using restricted cubic spline regression. The analysis adjusted for gestational age (modeled as a continuous variable and shown on the *x*‐axis), birth weight (fixed at the mean), and ACS administration (fixed at no administration). The solid line represents the adjusted estimate, and the shaded area indicates the 95% confidence interval. The horizontal line at *y* = 0 indicates the reference level.

RCS visualization treating gestational age as a continuous variable revealed that the adjusted estimates and 95% CI for both magnesium sulfate administration (Figure 3a) and high magnesium levels (Figure 3b) were above zero after 30 weeks of gestation, with a peak around 32 weeks.

## 4 Discussion

This study revealed that magnesium sulfate administration was significantly associated with higher S100B levels at 30 weeks of gestation and later, but did not before 30 weeks at delivery. It was also shown that the effects of magnesium sulfate administration and magnesium levels on S100B levels depend on gestational age.

Several studies involving children and adults have demonstrated that magnesium sulfate administration can suppress the rise in S100B levels during critical events such as hypoxic–ischemic encephalopathy (HIE), brain surgery, and stroke [24, 25, 26]. Based on the available evidence, it was expected that magnesium sulfate administration would lower cord serum S100B levels; however, magnesium sulfate administration was associated with increased S100B levels in the ≥ 30 weeks group in all regression models. As shown by the RCS analysis (Figure 3), the association between magnesium sulfate administration and S100B levels peaked at approximately 32 weeks of gestation and subsequently attenuated beyond 33–34 weeks. This pattern suggests the presence of a limited gestational window during which the fetal brain may respond differently to antenatal magnesium sulfate exposure. Human cortical development remains highly dynamic during late gestation (approximately ≥ 28 weeks of gestation). At this point, ongoing cortical organization and lamination, synaptogenesis and large‐scale network formation [27, 28], peak activity of the transient fetal subplate [27, 29], astrocyte maturation [30], progressive development of BBB function [31], glymphatic processes, and developmentally distinct fetal microglial activity [32] are observed. These neurodevelopmental processes may create a gestational window in which the fetal brain is particularly responsive to exogenous modulation, including antenatal magnesium sulfate exposure. Therefore, the attenuation of the effect of magnesium sulfate on S100B levels after 32 weeks in our analysis may reflect continued maturation of these fetal mechanisms. Further validation using experimental models and complementary approaches will be required to clarify the biological significance of this gestationally restricted phenomenon.

In our review, we identified only two studies examining the association between antenatal magnesium sulfate and S100B levels. One case–control study compared patients with cerebral palsy or neonatal death to those without cerebral palsy, focusing on neonates born between 24 and 31 weeks of gestation [9]. In a subanalysis of this study, cord S100B levels were compared between neonates who received antenatal magnesium sulfate and those who received placebo, in both cases and controls. However, no significant difference in cord S100B levels was observed. This finding is consistent with our results in the < 30 weeks at delivery group. Magnesium sulfate may not affect cord serum S100B levels before 32 weeks of gestation. Another study was an animal experiment investigating S100B expression in the fetal brain using two preterm birth models [33]. Magnesium sulfate administration, compared with normal saline, significantly reduced S100B levels in the fetal brain in the inflammation‐induced preterm birth model, but not in a non‐inflammatory preterm birth model. The speculation from this animal study is that antenatal magnesium sulfate may have neuroprotective effects in highly inflammatory conditions but may not provide the same benefit in cases of preterm birth without inflammation. However, it should be noted that embryonic 15 days in mice corresponds to approximately 11 weeks of gestation in human brain development [34]. Therefore, the result of this study cannot be directly extrapolated to human preterm birth. The present study also included some h‐CAM patients, but did not show a decreasing effect of S100B levels by magnesium sulfate.

We also conducted multiple regression analysis with models including h‐CAM or IL‐6. IL‐6 was known as a marker of fetal inflammation and used to diagnose fetal inflammatory response syndrome [35]. As a result, we showed no correlation between IL‐6 and S100B levels, but showed a significant correlation between h‐CAM and S100B in the ≥ 30 gestation group. This is consistent with a previous observational study that showed higher S100B concentration in intrauterine infection cases than those in non‐infected controls, including patients with 34.2–34.7 weeks of mean gestational age at delivery [36]. In addition, ACS was significantly associated with lower S100B levels in all regression models. This result is consistent with previous reports [20, 37]. Both antenatal corticosteroids and magnesium sulfate have neuroprotective effects in preterm infants; however, their opposing effects on cord serum S100B levels highlight the differences in their underlying mechanisms.

The strength of this study is as follows. First, to the best of our knowledge, this is the first study which mainly investigated the association between antenatal magnesium sulfate administration and cord serum S100B levels. Second, the significant association between magnesium sulfate and cord serum S100B levels was robust in multiple sensitivity analyses.

This study had several limitations. First, it was a retrospective study; therefore, the possibility of potential confounding factors could not be completely ruled out. However, we attempted to minimize confounding by employing multivariate analysis and excluding cases with HDP and category 1 cesarean births. Second, in the MgSO_4_ group, only 66.7% of cases before 30 weeks of gestation had umbilical cord magnesium concentrations exceeding the 90th percentile of the control group. Since this study excluded patients with HDP, the primary indications for magnesium sulfate administration were fetal neuroprotection or maintenance tocolysis, and specific target maternal serum magnesium levels were not consistently established. This variability may have contributed to the inconsistent significance between magnesium sulfate and higher magnesium levels observed in interaction analysis by gestational age groups. Third, the limited sample size made it difficult to identify specific patient characteristics associated with greater increases in S100B levels following magnesium sulfate administration.

Our results suggest that magnesium sulfate administration beyond 30 weeks of gestation has some effect on fetal brain. We could not conclude this is harmful; however, our findings are consistent with the secondary analysis of the MAGENTA trial, which speculated a potential adverse impact of magnesium sulfate at this gestation on offspring's neurodevelopment at 2 years of age.

In conclusion, magnesium sulfate administration was significantly associated with higher cord serum S100B levels in neonates born at 30^+0^–33^+6^ weeks of gestation, but not in those born at 22^+0^–29^+6^ weeks. This association could be specific for gestational week. Further research is needed to clarify whether antenatal magnesium sulfate has any adverse effects on the fetal brain when administered at ≥ 30 weeks of gestation.

## Author Contributions

**Takuma Shimaya:** conceptualization, investigation, writing – original draft. **Kazuya Fuma:** conceptualization, investigation, data curation, visualization, writing – original draft, funding acquisition. **Sho Tano:** investigation, writing – review and editing. **Seiko Matsuo:** investigation, writing – review and editing. **Takafumi Ushida:** conceptualization, investigation, project administration, writing – review and editing. **Kenji Imai:** writing – review and editing. **Hiroaki Kajiyama:** writing – review and editing, supervision. **Tomomi Kotani:** funding acquisition, writing – review and editing, supervision.

## Funding

This work was supported by Japan Society for the Promotion of Science, 22K09638, 24K23509.

## Disclosure

Dr. Hiroaki Kajiyama is the Editor‐in‐Chief of the journal and the co‐author of this article. They were excluded from the peer‐review process and all editorial decisions related to the acceptance and publication of this article. Peer review was handled independently by the JOGR Journal editorial office to minimize bias.

## Ethics Statement

This study was carried out following The Code of Ethics of the World Medical Association (Declaration of Helsinki), and Uniform Requirements for manuscripts submitted to Biomedical journals. The Institutional Ethics Board of Nagoya University Hospital approved this study protocol (approval number: 2018‐0211, 2018‐0026).

## Consent

Written informed consent was obtained from the study participants when collecting umbilical cord blood samples for the study.

## Conflicts of Interest

The authors declare no conflicts of interest.

## Supporting information


**Table S1:** Effects of cord serum magnesium level on S100B levels stratified by gestational age (< 30 weeks at delivery vs. ≥ 30 weeks at delivery).
**Raw data**. Background characteristics and cord serum raw data of 69 patients.

## Data Availability

The data that supports the findings of this study are available in the Supporting Information of this article.
