# Supplementary material for: Gestational Age‐Dependent Effects of Antenatal Magnesium Sulfate on Fetal S100B Levels: An Observational Study Using Cord Serum
Source: J Obstet Gynaecol Res. 2026 Feb 15;52(2):e70208. doi: 10.1111/jog.70208 (PMC12907285; doi:10.1111/jog.70208)
Supplement: Supplementary file 1 — Table S1: Effects of cord serum magnesium level on S100B levels stratified by gestational age (< 30 weeks at delivery vs. ≥ 30 weeks at delivery). Raw data. Background characteristics and cord serum raw data of 69 patients. [file JOG-52-0-s001.zip › jog70208-sup-0002-Supinfo2@Supplementary_Table.docx]

Supplementary table. Effects of Cord Serum Magnesium Level on S100B Levels Stratified by Gestational Age (<30 weeks vs ≥30 weeks)

|  | Cord serum S100B levels (ng/mL) | | | | | | | | | | | | | |
| --- | --- | --- | --- | --- | --- | --- | --- | --- | --- | --- | --- | --- | --- | --- |
|  | Crude, B (95% CI) | |  | Model 1, B (95% CI) | |  | Model 2, B (95% CI) | |  | Model 3, B (95% CI) | |  | Model 4, B (95% CI) | |
|  | <30 weeks | ≥30 weeks |  | <30 weeks | ≥30 weeks |  | <30 weeks | ≥30 weeks |  | <30 weeks | ≥30 weeks |  | <30 weeks | ≥30 weeks |
|  | n = 24 | n = 45 |  | n = 24 | n = 45 |  | n = 24 | n = 45 |  | n = 24 | n = 45 |  | n = 23^a^ | n = 43^a^ |
| Variable |  |  |  |  |  |  |  |  |  |  |  |  |  |  |
| Mg > 90th percentile^b^ | 0.08 (−0.30, 0.47) | 0.37 (0.12, 0.62)** |  | 0.13 (−0.19, 0.44) | 0.35 ( 0.13, 0.58)** |  | 0.18 (−0.20, 0.55) | 0.35 ( 0.12, 0.58)** |  | 0.16 (−0.17, 0.48) | 0.28 ( 0.05, 0.50)* |  | 0.10 (−0.23, 0.43) | 0.28 ( 0.04, 0.52)* |
| Covariates |  |  |  |  |  |  |  |  |  |  |  |  |  |  |
| Birth weight (kg) |  |  |  | 0.04 (−0.51, 0.59) | −0.31 (−0.58, −0.05)* |  | 0.02 (−0.54, 0.59) | −0.32 (−0.59, −0.05)* |  | −0.05 (−0.63, 0.54) | −0.37 (−0.63, −0.11)** |  | −0.02 (−0.63, 0.58) | −0.37 (−0.65, −0.10)** |
| ACS |  |  |  | −0.71 (−1.12, −0.29)** | −0.31 (−0.53, −0.09)** |  | −0.67 (−1.12, −0.23)** | −0.31 (−0.53, −0.08)** |  | −0.69 (−1.11, −0.27)** | −0.36 (−0.58, −0.14)** |  | −0.83 (−1.31, −0.35)** | −0.38 (−0.61, −0.14)** |
| Labor onset |  |  |  |  |  |  | −0.10 (−0.51, 0.31) | 0.03 (−0.17, 0.24) |  |  |  |  |  |  |
| h-CAM |  |  |  |  |  |  |  |  |  | −0.17 (−0.54, 0.20) | 0.27 ( 0.03, 0.52)* |  |  |  |
| IL-6 [log-transformed] |  |  |  |  |  |  |  |  |  |  |  |  | 0.00 (−0.06, 0.06) | 0.03 ( 0.00, 0.06) |
| R*^2^* | 0.01 | 0.17 |  | 0.41 | 0.39 |  | 0.41 | 0.39 |  | 0.43 | 0.46 |  | 0.46 | 0.44 |
| F | F(1, 22) = 0.20 | F(1, 43) = 8.96** |  | F(3, 20) = 4.56* | F(3, 41) = 8.74*** |  | F(4, 19) = 3.37* | F(4, 40) = 6.43*** |  | F(4, 19) = 3.64* | F(4, 40) = 8.50*** |  | F(4, 18) = 3.76* | F(4, 38) = 7.37*** |
| p-value for the interaction between gestational groups and MgSO4 estimate |  | 0.18 |  |  | 0.16 |  |  | 0.19 |  |  | 0.18 |  |  | 0.2 |

The effects of high cord magnesium levels (>90^th^ percentile of the control group) on cord serum S100B levels, stratified by gestational age (<30 weeks vs. ≥30 weeks). Model 1: Adjusted for birth weight and ACS. Model 2: Adjusted for birth weight, ACS, and labor onset. Model 3: Adjusted for birth weight, ACS, and h-CAM. Model 4: Adjusted for birth weight, ACS, and log-transformed IL-6 level. ^a^Number reduction due to limited sample. ^b^Higher than 90^th^ percentile in the control group (2.56 mg/dL). CI: confidence interval, ACS: antenatal corticosteroids, h-CAM: histological chorioamnionitis. *p <0.05. **p <0.01. ***p <0.001.
